# Analysis of EGFR, KRAS, and PIK3CA gene mutation rates and clinical distribution in patients with different types of lung cancer

**DOI:** 10.1186/s12957-021-02315-1

**Published:** 2021-07-03

**Authors:** Shuo Li, Xinju Li

**Affiliations:** Department of Thoracic Surgery, The First Affiliated Hospital of Xi’an Jiaotong University, Xi’an, 710061 Shaanxi Province China

**Keywords:** Lung cancer, Gene mutation, EGFR, KRAS, PIK3CA, Clinic

## Abstract

**Background:**

To analyze and evaluate EGFR, KRAS, and PIK3CA gene mutation rates and clinical distribution in patients with different types of lung cancer

**Method:**

A total of 221 lung cancer patients treated in our hospital between January 2016 and June 2019 were enrolled. Tissue and whole blood samples were collected and analyzed to determine the mutation status of EGFR, KRAS, and PIK3CA genes. The gene exon mutation rates were determined. Relevant clinical data, such as age, gender, tumor sample type, treatment method, pathologic type, and lung cancer stage were recorded and statistically analyzed.

**Results:**

The EGFR gene mutation rates in exons E18-E21 were 2.3%, 17.6%, 3.6%, and 20.4%, respectively. E18, E19, and E20 mutations were commonly detected in adenosquamous carcinoma, and E21 mutations were commonly detected in adenocarcinoma. Mutations in exons E18-E21 were frequently detected in patients with lung cancer stages IA, IB, IIA, or IIB, respectively. The KRAS gene mutation rate in lung cancer patients in exon E2 was higher in whole blood and tissue samples than other exon mutations, while the KRAS gene mutation rate in exons E2 and E3 was significantly higher in patients with lung cancer stages IIB and IA, respectively. PIK3CA gene mutations in exons E9 and E20 occurred in patients < 60 years of age. Exon E9-positive mutations were more common in men or patients with squamous cell carcinoma, while exon E20-positive mutations were more common in females.

**Conclusion:**

The EGFR, KRAS, and PIK3CA gene exon mutation rates differ and were shown to be correlated with different clinical indicators, which have significance in clinical treatment.

## Background

Lung cancer is the second most commonly diagnosed malignant cancer, with an incidence of 11.4% among all new cancer cases [1]. Lung cancer remains the leading cause of cancer deaths, with an estimated 1.8 million deaths annually [1]. Non-small cell lung cancer (NSCLC), including adenocarcinoma, squamous cell carcinoma, large cell carcinoma, adenosquamous carcinoma, sarcomatoid carcinoma, and mucoepidermoid carcinoma, accounts for 75–80% of the total number of lung cancer cases [2]. Moreover, the prognosis is poor, and the 5-year survival rate is < 20% [2]. The mainstay of treatment for stage I-IIIa NSCLC patients is surgical resection with adjuvant chemotherapy. The National Comprehensive Cancer Network (NCCN) clinical guidelines for NSCLC recommend adjuvant chemotherapy for stage Ib-IIIa NSCLC patients after surgery [3]; however, due to individual differences and drug resistance, the effect of adjuvant chemotherapy differs. Therefore, individualized treatment has been proposed to achieve higher success rates than standardized treatment [4].

With the rapid advances in modern molecular biology technology, the treatment model for lung cancer has focused on targeting abnormal molecules in specific signaling pathways [5, 6]. In the past 10 years, tyrosine kinase inhibitors (TKIs) for epidermal growth factor receptor (EGFR) have demonstrated remarkable clinical effects and paved the way for effective treatment of lung cancer [7–9]. Indeed, a recent phase III clinical trial (ADAURA [NCT02511106]) assessed the efficacy and safety of a 3rd-generation EGFR-TKI, osimertinib, which had superior efficacy when compared to EGFR-TKI (gefitinib/erlotinib) in treatment-naïve patients with EGFR-mutated advanced NSCLC [10]. In addition, rapid and accurate determination of EGFR gene mutation status is important to correctly adjust the chemotherapy regimen and usage of drugs. In recent years, with the ongoing in-depth studies of molecular biology and human genomics, it has been shown that EGFR-TKI treatment is effective in NSCLC patients with E19/21 EGFR mutations along with the metastases, and these patients had a longer progression-free survival (PFS) [11]. EGFR-TLI treatment of patients with KRAS mutations lacks efficacy, which indicates the different patterns of EGFR and KRAS gene mutations in lung cancer patients.

Phosphatidylinositol-3 kinase (PIK3CA) is a coding gene for the protein catalytic subunit of the phosphatidylinositol-3 kinase family (PI3Ks) [12]. It has been well-documented that activation of the PI3Ks pathway is involved in multiple human malignancies, while the effect of PIK3CA mutations on the prognosis of patients is controversial for different human cancers. It has been demonstrated that PIK3CA mutation status predicts the prognosis of breast cancer patients [13], while another study that PIK3CA mutation status is not associated with prognosis of colon cancer patients [14]. Few studies have systematically evaluated the relationship between the mutation status of EGFR, KRAS, and PIK3CA and lung cancer patients, especially among patients with different types of lung cancer.

In this study, we analyzed the mutation status of EGFR, KRAS, and PIK3CA in different types of lung cancer patients. These findings may provide theoretical insight for clinicians to make accurate and instant treatment plans for lung cancer patients.

## Materials and methods

### General information

A total of 221 lung cancer patients (114 males [51.6%] and 107 females [48.4%]) who were treated in our hospital from January 2016 to June 2019 were enrolled in the current study. The following specimens were collected: 194 tissue samples (87.7%), 22 whole blood samples (10.0%), and 5 whole blood + tissue samples (2.3%). One hundred twelve patients (50.7%) were ≤ 60 years of age, 109 (49.3%) were > 60 years of age, and the average age was 59.62 ± 9.82 years. The clinical characteristics of the patients are presented in Table 1. All paraffin-embedded tissue sections and cytologic smears were examined and diagnosed by senior pathologists. The tumor areas were delineated, and the tumor cell number (> 200) and percentage (> 20%) were evaluated. Ten milliliters of peripheral venous blood samples was collected in blood collection tubes dedicated to protect free DNA for normal temperature transportation and preservation.

**Table 1:** Clinical characteristics of patients enrolled in this study

Characteristics		Number (%)
**Age (year)**	59.62 ± 9.82	221
	≤60	112 (50.7)
	>60	109 (49.3)
**Gender**	Male	114 (51.6)
	Female	107 (48.4)
**Pathological type**	Squamous cell carcinoma	15 (6.8)
	Adenocarcinoma	155 (70.1)
	Adenosquamous carcinoma	7 (3.2)
	Others	44 (19.9)
**Staging**	Stage IA	22 (10.0)
	Stage IB	51 (23.1)
	Stage IIA	8 (3.6)
	Stage IIB	9 (4.1)
	Stage IIIA	23 (10.4)
	Stage IIIB	5 (2.3)
	Stage IV	33 (14.9)
	Undefined	70 (31.7)
Treatment	Surgery	67 (30.3)
	Chemotherapy	37 (16.7)
	Surgery + chemotherapy	66 (30.0)
	Not available	51 (23.0)

### Reagents

Formaldehyde-fixed, paraffin-embedded tissue DNA extraction kits were purchased from Qiagen Company (Darmstadt, Germany). Tissue DNA extraction kits, plasma circulating DNA extraction kits, human EGFR gene mutation detection kits (ARMS method [15]), and human EGFR, KRAS, and PIK3CA gene mutation detection kits (super-ARMS method [16]) were purchased from AmoyDx (Xiamen, China). An ABI fluorescence quantitative PCR instrument (Foster city, CA, USA) was used.

### Detection of EGFR, KRAS, and PIK3CA gene mutations

Sample processing and DNA extraction were performed according to the manufacturer’s protocol. The DNA extraction protocol from paraffin-tissue samples was as follows: refrigerate the circled wax block and place the block on a slicer, cut the samples into 10 pieces (4-μm-thick slices), remove the non-cancerous tissue, and transfer the wax into a 2-ml centrifuge tube. Extract 50 μl of DNA according to the formaldehyde-fixed, paraffin-embedded tissue DNA extraction kit protocol. The DNA extraction from peripheral blood sample protocol was as follows: collect the samples and centrifuge the samples immediately at 2000×*g* for 10 min, aspirate the supernatant and transfer the sample to a new centrifuge tube, centrifuge the samples at 8000×*g* for 10 min, and aspirate the supernatant and transfer to a new centrifuge tube. PBS was added to the tube if the volume was < 4 ml, and 100 μl of DNA was extracted according to the plasma circulating DNA extraction kit protocol.

The EGFR (E18, E19, E20, and E21), KRAS (E2, E3, and E4), and PIK3CA (E9 and E20) gene mutations in lung cancer samples were detected using liquid chip technology. The main research steps were as follows: the gene fragments containing common alleles in the exons of EGFR, KRAS, and PIK3CA genes were obtained using multiplex PCR; the reaction products were hydrolyzed by an exonuclease and alkaline phosphatase (EXO-SAP); the allele-specific primer extension (ASPE) was used for PCR product processing; the tag sequence on the amplified product and anti-tag sequence on polystyrene microspheres were specifically hybridized; and the hybridized product was analyzed using a Luminex 200 system (Austin, TX, USA) to obtain the median fluorescence value (MFI).

### Statistical analysis

The SPSS 19.0 software was used for statistical analyses. The correlation between EGFR, KRAS, and PIK3CA gene mutations and clinicopathologic features of patients was analyzed using unpaired χ^2^ tests or Fisher exact probability analysis [17]. The difference was statistically significant (p< 0.05).

## Results

### Detection and analysis of EGFR gene mutations

As shown in Table 2, mutation of the EGFR gene in exon E18 was significantly related to sample type, pathologic type, lung cancer stage, and treatment method. The E18 mutation was mainly detected in tissue samples (2.58%) and adenosquamous carcinoma (14.29%). In addition, stage IA lung cancer patients (13.64%) and patients who underwent surgical resection (5.97%) were diagnosed with a higher E18 mutation rate; however, there was no significant difference between the E19 and E20 mutation status and clinical indicators (Tables 3 and 4). The E19-positive mutation rate was higher in adenosquamous carcinoma (25.49%) and stage IB patients (28.57%), while the E20-positive mutation rate was higher in adenosquamous carcinoma (14.29%) and stage IIIB stage patients (20.00%).

**Table 2:** Relationship between mutation of EGFR gene in exon E18 and clinical parameters

	Clinical parameters	No.	EGFR-E18		Positive rate (%)	χ^2^	p value
			Wild type	Mutant type			
Age	≤60	112	93	1	0.89	4.067	0.131
	>60	109	95	4	3.67		
Gender	Male	114	97	1	0.88	2.344	0.310
	Female	107	91	4	3.74		
Sample type	Tissue sample	194	178	5	2.58	73.377	**<0.001**
	Whole blood sample	22	7	0	0		
	Whole blood + tissue sample	5	3	0	0		
Pathological type	Squamous cell carcinoma	15	10	0	0	16.489	**0.011**
	Adenocarcinoma	155	138	4	2.58		
	Adenosquamous carcinoma	7	5	1	14.29		
Staging	Stage IA	22	19	3	13.64	40.276	**<0.001**
	Stage IB	51	48	1	1.96		
	Stage IIA	8	7	1	12.50		
	Stage IIB	9	8	0	0		
	Stage IIIA	23	20	0	0		
	Stage IIIB	5	2	0	0		
	Stage IV	33	28	0	0		
Treatment	Surgery	67	61	4	5.97	20.081	**0.003**
	Chemotherapy	37	28	0	0		
	Surgery + chemotherapy	66	59	1	1.52		

Data are adjusted for age, gender, sample type, pathological type, staging, and treatment method

**Table 3:** Relationship between mutation of EGFR gene in exon E19 and clinical parameters

	Clinical parameters	No.	EGFR-E19		Positive rate (%)	χ^2^	p value
			Wild type	Mutant type			
Age	≤60	112	85	24	21.43	2.241	0.326
	>60	109	91	15	13.76		
Gender	Male	114	96	14	12.28	5.007	0.082
	Female	107	80	25	23.36		
Sample type	Tissue sample	194	155	34	17.53	2.020	0.732
	Whole blood sample	22	16	5	22.73		
	Whole blood + tissue sample	5	5	0	0		
Pathological type	Squamous cell carcinoma	15	14	0	0	10.285	0.113
	Adenocarcinoma	155	125	28	18.06		
	Adenosquamous carcinoma	7	4	2	28.57		
Staging	Stage IA	22	18	4	18.18	20.438	0.117
	Stage IB	51	38	13	25.49		
	Stage IIA	8	8	0	0		
	Stage IIB	9	7	1	11.11		
	Stage IIIA	23	19	2	8.70		
	Stage IIIB	5	4	0	0		
	Stage IV	33	28	5	15.15		
Treatment	Surgery	67	52	14	20.90	3.313	0.769
	Chemotherapy	37	31	5	13.51		
	Surgery + chemotherapy	66	54	9	13.64		

Data are adjusted for age, gender, sample type, pathological type, staging, and treatment method

**Table 4:** Relationship between mutation of EGFR gene in exon E20 and clinical parameters

	Clinical parameters	No.	EGFR-E20		Positive rate (%)	χ^2^	p value
			Wild type	Mutant type			
Age	≤60	112	104	5	4.46	1.538	0.463
	>60	109	100	3	2.75		
Gender	Male	114	103	4	3.51	2.578	0.276
	Female	107	101	4	3.74		
Sample type	Tissue sample	194	178	8	4.12	1.392	0.845
	Whole blood sample	22	21	0	0		
	Whole blood + tissue sample	5	5	0	0		
Pathological type	Squamous cell carcinoma	15	13	0	0	9.188	0.163
	Adenocarcinoma	155	146	5	3.23		
	Adenosquamous carcinoma	7	5	1	14.29		
Staging	Stage IA	22	22	0	0	17.414	0.235
	Stage IB	51	48	2	3.92		
	Stage IIA	8	7	1	12.50		
	Stage IIB	9	8	0	0		
	Stage IIIA	23	21	0	0		
	Stage IIIB	5	3	1	20.00		
	Stage IV	33	32	1	3.03		
Treatment	Surgery	67	63	2	2.99	2.131	0.907
	Chemotherapy	37	34	1	2.70		
	Surgery + chemotherapy	66	59	4	6.06		

Data are adjusted for age, gender, sample type, pathological type, staging, and treatment method

As shown in Table 5, mutation of the EGFR gene in exon E21 was significantly associated with gender, lung cancer stage, and treatment method. Specifically, the E21-positive mutation rate in females (29.91%) was significantly higher than males (11.40%), and the E21 mutation rate in patients with stage IB lung cancer (33.33%) was significantly higher than other stages. Like the E18 mutation rate, the patients who underwent surgical resection (37.31%) had a higher E18 mutation rate than patients who were treated with chemotherapy (10.81%) or surgical resection combined with chemotherapy (13.64%). Even though there was no significant difference between pathologic type and the E21-positive mutation rate, the results showed that the patients with adenocarcinoma had the highest E21 mutation rate (24.52%).

**Table 5:** Relationship between mutation of EGFR gene in exon E21 and clinical parameters

	Clinical parameters	No.	EGFR-E21		Positive rate (%)	χ^2^	p value
			Wild type	Mutant type			
Age	≤60	112	84	24	21.43	0.159	0.923
	>60	109	84	21	19.27		
Gender	Male	114	96	13	11.40	11.741	**0.003**
	Female	107	72	32	29.91		
Sample type	Tissue sample	194	142	45	23.20	8.171	0.086
	Whole blood sample	22	21	0	0		
	Whole blood + tissue sample	5	5	0	0		
Pathological type	Squamous cell carcinoma	15	14	0	0	9.627	0.141
	Adenocarcinoma	155	113	38	24.52		
	Adenosquamous carcinoma	7	5	1	14.29		
Staging	Stage IA	22	15	7	31.82	26.220	**0.024**
	Stage IB	51	33	17	33.33		
	Stage IIA	8	7	1	12.50		
	Stage IIB	9	8	0	0		
	Stage IIIA	23	14	7	30.43		
	Stage IIIB	5	4	0	0		
	Stage IV	33	27	6	18.18		
Treatment	Surgery	67	40	25	37.31	18.020	**0.006**
	Chemotherapy	37	31	4	10.81		
	Surgery + chemotherapy	66	54	9	13.64		

Data are adjusted for age, gender, sample type, pathological type, staging, and treatment method

### Detection and analysis of KRAS gene mutations

As shown in Table 6, mutation of the KRAS gene in exon E2 was significantly related to sample type. In contrast to mutation of the EGFR gene in exon E18, the E2-positive KRAS mutation rate in the whole blood + tissue sample (20.00%) was significantly higher than the whole blood (0%) or tissue sample (7.22%). There was no significant difference between the E2 mutation rate and other clinical indicators. Patients with adenosquamous carcinoma (14.29%) and patients with stage IIB lung cancer (11.11%) had a higher E2 mutation rate in the KRAS gene. Similar to mutation of the EGFR gene in exons E20 and E21, there was no significant difference between the KRAS gene mutation rate in exon E3 and clinical indicators (Table 7). The E3-positive mutation rate was more common in adenocarcinoma patients (0.65%) and patients with stage IA lung cancer (4.55%). Interestingly, there were no positive mutations of the KRAS gene in exon E4 detected in samples collected from the current study (Table 8).

**Table 6:** Relationship between mutation of KRAS gene in exon E2 and clinical parameters

	Clinical parameters	No.	KRAS-E2		Positive rate (%)	χ^2^	p value
			Wild type	Mutant type			
Age	≤60	112	79	8	7.14	0.207	0.902
	>60	109	75	7	6.42		
Gender	Male	114	78	11	9.65	3.151	0.207
	Female	107	76	4	3.74		
Sample type	Tissue sample	194	129	14	7.22	10.462	**0.033**
	Whole blood sample	22	21	0	0		
	Whole blood + tissue sample	5	4	1	20.00		
Pathological type	Squamous cell carcinoma	15	12	0	0	5.415	0.492
	Adenocarcinoma	155	104	10	6.45		
	Adenosquamous carcinoma	7	4	1	14.29		
Staging	Stage IA	22	16	0	0	10.624	0.715
	Stage IB	51	33	5	9.80		
	Stage IIA	8	6	0	0		
	Stage IIB	9	7	1	11.11		
	Stage IIIA	23	16	0	0		
	Stage IIIB	5	4	0	0		
	Stage IV	33	27	2	6.06		
Treatment	Surgery	67	48	4	5.97	2.406	0.879
	Chemotherapy	37	25	1	2.70		
	Surgery + chemotherapy	66	45	6	9.09		

Data are adjusted for age, gender, sample type, pathological type, staging, and treatment method

**Table 7:** Relationship between mutation of KRAS gene in exon E3 and clinical parameters

	Clinical parameters	No.	KRAS-E3		Positive rate (%)	χ^2^	p value
			Wild type	Mutant type			
Age	≤60	112	85	1	0.89	0.075	0.963
	>60	109	81	1	0.92		
Gender	Male	114	87	1	0.88	0.183	0.913
	Female	107	79	1	0.93		
Sample type	Tissue sample	194	140	2	1.03	7.436	0.115
	Whole blood sample	22	21	0	0		
	Whole blood + tissue sample	5	5	0	0		
Pathological type	Squamous cell carcinoma	15	12	0	0	3.399	0.757
	Adenocarcinoma	155	113	1	0.65		
	Adenosquamous carcinoma	7	5	0	0		
Staging	Stage IA	22	15	1	4.55	9.357	0.808
	Stage IB	51	38	0	0		
	Stage IIA	8	6	0	0		
	Stage IIB	9	8	0	0		
	Stage IIIA	23	16	0	0		
	Stage IIIB	5	4	0	0		
	Stage IV	33	29	0	0		
Treatment	Surgery	67	51	1	1.49	2.612	0.856
	Chemotherapy	37	26	0	0		
	Surgery + chemotherapy	66	51	0	0		

Data are adjusted for age, gender, sample type, pathological type, staging, and treatment method

**Table 8:** Relationship between mutation of KRAS gene in exon E4 and clinical parameters

	Clinical parameters	No.	KRAS-E4		Positive rate (%)	χ^2^	p value
			Wild type	Mutant type			
Age	≤60	112	58	0	0	2.998	0.103
	>60	109	69	0	0		
Gender	Male	114	70	0	0	1.493	0.276
	Female	107	57	0	0		
Sample type	Tissue sample	194	120	0	0	15.433	**<0.001**
	Whole blood sample	22	4	0	0		
	Whole blood + tissue sample	5	3	0	0		
Pathological type	Squamous cell carcinoma	15	9	0	0	0.047	0.997
	Adenocarcinoma	155	89	0	0		
	Adenosquamous carcinoma	7	4	0	0		
Staging	Stage IA	22	13	0	0	8.274	0.309
	Stage IB	51	34	0	0		
	Stage IIA	8	5	0	0		
	Stage IIB	9	8	0	0		
	Stage IIIA	23	13	0	0		
	Stage IIIB	5	2	0	0		
	Stage IV	33	16	0	0		
Treatment	Surgery	67	45	0	0	8.807	**0.032**
	Chemotherapy	37	17	0	0		
	Surgery + chemotherapy	66	42	0	0		

Data are adjusted for age, gender, sample type, pathological type, staging, and treatment method

### Detection and analysis of PIK3CA gene mutations

As shown in Table 9, mutation of the PIK3CA gene in exon E9 was significantly related to the sample and pathologic types. The positive E9 mutation rates in the whole blood + tissue sample (20.00%) and patients with squamous cell carcinoma (13.33%) were the highest. In addition, E9-positive mutations were more common in stage IIB lung cancer patients (11.11%) and patients treated with chemotherapy (5.41%). The mutation status of the PIK3CA gene in exon E20 was significantly associated with sample type (Table 10). The rate of positive E20 mutations in the whole blood sample (4.55%) was significantly higher than the other two sample types; however, there were no positive PIK3CA gene mutations detected in exon E20 among patients with different pathologic types or lung cancer stages (Table 10).

**Table 9:** Relationship between mutation of PIK3CA gene in exon E9 and clinical parameters

	Clinical parameters	No.	PIK3CA-E9		Positive rate (%)	χ^2^	p value
			Wild type	Mutant type			
Age	≤60	112	82	3	2.68	1.087	0.581
	>60	109	79	1	0.92		
Gender	Male	114	82	4	3.51	3.838	0.147
	Female	107	79	0	0		
Sample type	Tissue sample	194	139	2	1.03	13.773	**0.008**
	Whole blood sample	22	18	1	4.55		
	Whole blood + tissue sample	5	4	1	20.00		
Pathological type	Squamous cell carcinoma	15	10	2	13.33	13.353	**0.038**
	Adenocarcinoma	155	112	1	0.65		
	Adenosquamous carcinoma	7	5	0	0		
Staging	Stage IA	22	16	0	0	10.663	0.712
	Stage IB	51	36	1	1.96		
	Stage IIA	8	6	0	0		
	Stage IIB	9	7	1	11.11		
	Stage IIIA	23	16	0	0		
	Stage IIIB	5	4	0	0		
	Stage IV	33	28	1	3.03		
Treatment	Surgery	67	52	0	0	5.112	0.530
	Chemotherapy	37	24	2	5.41		
	Surgery + chemotherapy	66	49	1	1.52		

Data are adjusted for age, gender, sample type, pathological type, staging, and treatment method

**Table 10:** Relationship between mutation of PIK3CA gene in exon E20 and clinical parameters

	Clinical parameters	No.	PIK3CA-E20		Positive rate (%)	χ^2^	p value
			Wild type	Mutant type			
Age	≤60	112	83	1	0.89	1.127	0.569
	>60	109	79	0	0		
Gender	Male	114	85	0	0	1.175	0.556
	Female	107	77	1	0.93		
Sample type	Tissue sample	194	139	0	0	12.794	**0.012**
	Whole blood sample	22	18	1	4.55		
	Whole blood + tissue sample	5	5	0	0		
Pathological type	Squamous cell carcinoma	15	12	0	0	4.735	0.578
	Adenocarcinoma	155	112	0	0		
	Adenosquamous carcinoma	7	5	0	0		
Staging	Stage IA	22	16	0	0	8.351	0.870
	Stage IB	51	36	0	0		
	Stage IIA	8	6	0	0		
	Stage IIB	9	8	0	0		
	Stage IIIA	23	16	0	0		
	Stage IIIB	5	4	0	0		
	Stage IV	33	29	0	0		
Treatment	Surgery	67	51	0	0	5.927	0.431
	Chemotherapy	37	25	1	2.70		
	Surgery + chemotherapy	66	50	0	0		

Data are adjusted for age, gender, sample type, pathological type, staging, and treatment method

## Discussion

The collective evidence has demonstrated that targeting abnormal genes in specific signaling pathways is a significant and effective strategy to eliminate cancers [18, 19]. In this study, we found that mutations of the EGFR gene in exons E18, E19, and E20 in lung cancer patients were common in adenosquamous carcinoma, while E21 mutations were common in adenocarcinoma. The E18 and E21 mutation rates were associated with lung cancer stage, gender, sample type, and treatment modality. Similarly, the KRAS gene mutation rate in exons E2 and E4 were also correlated with the sample type and treatment approach. In addition, the PIK3CA gene mutation status in exons E9 and E20 was associated with sample type, as well as lung cancer type. Taken together, these findings provide theoretical insight for clinicians to make accurate and timely treatment plans for lung cancer patients.

EGFR is a transmembrane tyrosine kinase receptor that plays an important role in the growth, proliferation, and differentiation of cells under physiologic and pathologic conditions. It has been reported that the EGFR mutation rate is 30–40% in Asian lung cancer patients [20] and 10–15% in European Caucasian patients [21]. Targeted therapies with EGFR-TKIs, such as gefitinib, erlotinib, and afatinib, have been well-established to effectively treat EGFR mutant NSCLC [22]; however, not all EGFR mutations are sensitive to EGFR-TKI treatment. For example, lung cancer cells with EGFR gene mutations in exons E19 and E21 are more sensitive to EGFR-TKI-mediated cell apoptosis, while cells with a mutation in exon 20 have increased resistance to EGFR-TKI-mediated cell apoptosis [23]. In addition, a recent 3rd-generation EGFR-TKI, osimertinib, had a statistically significant and clinically meaningful improvement in disease-free survival in patients with common sensitive EGFR-mutated NSCLC after complete tumor resection and adjuvant chemotherapy [10, 24]. Therefore, detection of EGFR mutation status in NSCLC patients before treatment is of great importance in the clinical setting. In this study, the positive EGFR mutation rates in exons E18-E21 were 2.3%, 17.6%, 3.6%, and 20.4% respectively. Mutations in exons E19 and E21 represented the vast majority (87%) of all observed EGFR gene mutations in lung cancer patients, which is consistent with another study [25]. Interestingly, the mutation rates in exons E18-E21 were significantly different in patients with stages IA, IB, IIA, or IIB, respectively. Nie et al. [26] reported that stage I lung adenocarcinoma patients had the highest incidence of mutations when compared with other pathologic stages [26], which was consistent with our finding. In addition, even though there was no statistical difference between the E19-E21 mutation status and patient gender or age, females and patients ≤ 60 years of age had a higher mutation rate than males, females, and patients > 60 years of age, which was in agreement with the dominant population of EGFR mutations in females [24] and young patients [27].

KRAS mutations are key oncogenic regulators in the development of many human malignant cancers, including lung cancer. KRAS mutations regulate signal transduction networks, which are necessary for cell survival, proliferation, differentiation, and apoptosis [28, 29]. Mutated KRAS leads to phosphorylation and activation of p21. Approximately 29% of NSCLC patients have KRAS gene mutations, among whom those with adenocarcinoma and a history of smoking are the most commonly diagnosed [30]. Collective evidence has demonstrated that the KRAS gene mutation status serves as a biomarker to predict the prognosis of NSCLC patients after chemotherapy [31]; however, KRAS and EGFR mutations are mutually exclusive, and NSCLC patients with KRAS mutations have a low response rate to EGFR-TKIs [32]. At present, several KRAS-targeting drugs, such as heat shock protein 90 (Hsp90) inhibitors, have been used in the treatment of NSCLC patients with KRAS-positive mutations with significant efficacy [33]. Therefore, except for detection of the EGFR gene, confirmation of KRAS gene mutation status is also important in the clinical setting.

In this study, it was shown that the positive KRAS gene mutation rate in exon E2 was highest in whole blood + tissue samples, patients with stage IIB, and patients with adenosquamous carcinoma; however, patients with adenocarcinoma or stage IA lung cancer were significantly more frequent than other lung cancer types or stages. Notably, no E4-positive mutations were detected in this study, which might reflect the limited sample size.

PI3Ks play a vital role in many biological processes and can activate serine/threonine kinase Akt and the downstream mTOR pathway to regulate cell survival, proliferation, and the cell cycle. Somatic mutations in the PI3K-Akt-mTOR pathway are often found in cancer tumorigenesis and can be used as a target for treatment of cancer patients [34]. PI3Ks consist of catalytic (P110) and regulatory subunits (p85), while catalytic subunits contain 3 gene codes (PIK3CA, PIK3CB, and PIK3CD). Generally, PIK3CA is the most common mutation location in cancer patients [35]. The most common mutation sites of PIK3CA are located in the spiral domain, including E542K and E545K in exon 9, and in the kinase domain, including H1047R in exon 20 [36]. In this study, we found that PIK3CA-E9 and the E20-positive mutation rates were higher in patients ≤ 60 years of age. E9-positive mutations were more common in males, while E20-positive mutations were more common in females. In addition, the positive PIK3CA-E9 mutation rate was highest in whole blood + tissue samples and patients with squamous cell carcinoma, while the E20 mutation was highest in whole blood samples.

The current study showed that the positive mutation rates in EGFR, KRAS, and PIK3CA gene exon loci were different which had a correlation with different clinical indicators. These findings may provide significance for the direction and predictability of clinical lung cancer treatment. With respect to the diagnosis and treatment of lung cancer, further understanding of these biomarkers can be used to assess the risk of tumor occurrence, early diagnosis, formulation of treatment plan, and evaluation of patient prognosis. In recent years, because the importance of biomarkers has been gradually revealed by scientists, more and more new biomarkers have been investigated and applied in the clinical setting. Therefore, better illustrating the significance of each biomarker in the diagnosis, treatment, and prognosis of lung cancer could potentially reduce the mistakes made in the early diagnosis of cancer patients and improve the efficacy of personalized treatment plans for cancer patients.

There were some limitations in this study. First, this was a single-center study with a small size of patients with lung cancer. Second, there were few patients with KRAS gene mutations in exons E3 (n=2) and E4 (n=0) or with PIK3CA gene mutations in exon E20 (n=1), which may result in inaccurate conclusions for these cohorts. Therefore, further investigations with a large sample size, as well as a comparison of samples in multiple centers, are needed to confirm the current findings. Third, the comparison among the different mutations and the relationship with clinical characteristics was not performed. Finally, the mechanism underlying these gene mutations and the correlation with clinical indicators, especially patient staging, are not fully elucidated, which requires further in vitro and in vivo studies.

## Conclusions

EGFR, KRAS, and PIK3CA gene mutations have a correlation with the clinical characteristics of lung cancer patients, which should be further accepted and improved to enhance the efficacy for personalized cancer treatment.

## Data Availability

The data and materials used or analyzed during the current study are available from the corresponding author upon reasonable request.

## Abbreviations

NSCLC: Non-small cell lung cancer
TKI: Tyrosine kinase inhibitor
EGFR: Epidermal growth factor receptor
PFS: Progression-free survival
PIK3CA: Phosphatidylinositol-3 kinase
PI3Ks: Phosphatidylinositol-3 kinase family
EXO-SAP: Exonuclease and alkaline phosphatase
ASPE: Allele-specific primer extension
MFI: Median fluorescence value
Hsp90: Heat shock protein 90
